# Engineering a Bifunctional Fusion Purine/Pyrimidine Nucleoside Phosphorylase for the Production of Nucleoside Analogs

**DOI:** 10.3390/biom14091196

**Published:** 2024-09-23

**Authors:** Daniel Hormigo, Jon Del Arco, Javier Acosta, Maximilian J. L. J. Fürst, Jesús Fernández-Lucas

**Affiliations:** 1 Applied Biotechnology Group, Universidad Europea de Madrid, Urbanización El Bosque, Villaviciosa de Odón, 28670 Madrid, Spain; daniel.hormigo@universidadeuropea.es (D.H.); jon.delarco@universidadeuropea.es (J.D.A.); javier.acosta@universidadeuropea.es (J.A.); 2 Molecular Enzymology Group, University of Groningen, Feringa Building, 9747 AG Groningen, The Netherlands; 3 Grupo de Investigación en Ciencias Naturales y Exactas, GICNEX, Universidad de la Costa, CUC, Calle 58 #55-66, Barranquilla 080002, Colombia; 4 Department of Biochemistry and Molecular Biology, Faculty of Biology, Universidad Complutense de Madrid, C. de José Antonio Novais, 12, 28040 Madrid, Spain

**Keywords:** biocatalysis, nucleoside analogs, nucleoside phosphorylases, fusion enzymes

## Abstract

Nucleoside phosphorylases (NPs) are pivotal enzymes in the salvage pathway, catalyzing the reversible phosphorolysis of nucleosides to produce nucleobases and α-D-ribose 1-phosphate. Due to their efficiency in catalyzing nucleoside synthesis from purine or pyrimidine bases, these enzymes hold significant industrial importance in the production of nucleoside-based drugs. Given that the thermodynamic equilibrium for purine NPs (PNPs) is favorable for nucleoside synthesis—unlike pyrimidine NPs (PyNPs, UP, and TP)—multi-enzymatic systems combining PNPs with PyNPs, UPs, or TPs are commonly employed in the synthesis of nucleoside analogs. In this study, we report the first development of two engineered bifunctional fusion enzymes, created through the genetic fusion of purine nucleoside phosphorylase I (PNP I) and thymidine phosphorylase (TP) from *Thermus thermophilus*. These fusion constructs, PNP I/TP-His and TP/PNP I-His, provide an innovative one-pot, single-step alternative to traditional multi-enzymatic synthesis approaches. Interestingly, both fusion enzymes retain phosphorolytic activity for both purine and pyrimidine nucleosides, demonstrating significant activity at elevated temperatures (60–90 °C) and within a pH range of 6–8. Additionally, both enzymes exhibit high thermal stability, maintaining approximately 80–100% of their activity when incubated at 60–80 °C over extended periods. Furthermore, the transglycosylation capabilities of the fusion enzymes were explored, demonstrating successful catalysis between purine (2′-deoxy)ribonucleosides and pyrimidine bases, and vice versa. To optimize reaction conditions, the effects of pH and temperature on transglycosylation activity were systematically examined. Finally, as a proof of concept, these fusion enzymes were successfully employed in the synthesis of various purine and pyrimidine ribonucleoside and 2′-deoxyribonucleoside analogs, underscoring their potential as versatile biocatalysts in nucleoside-based drug synthesis.

## 1. Introduction

Enzyme-mediated processes have emerged as efficient and environmentally sustainable alternatives for synthesizing nucleoside-based active pharmaceutical ingredients (APIs) [1,2,3,4,5,6]. Consequently, enzymatic or chemo-enzymatic approaches are progressively replacing chemical synthesis methods for the industrial production of nucleoside analogs [7,8].

Among existing biocatalytic alternatives, transglycosylation reactions catalyzed by nucleoside phosphorylases (NPs) [9,10] and 2′-deoxyribosyltransferases (NDTs) [11,12] stand out as pivotal components in many synthetic schemes for nucleoside synthesis. The utilization of enzyme-mediated transglycosylation reactions presents a highly efficient approach characterized by streamlined processes (e.g., one-pot reactions, reduced step count, enhanced atom economy), precise selectivity (both in terms of regioselectivity and stereoselectivity), and an environmentally conscientious pathway (owing to the absence of organic reagents and hazardous solvents) for nucleoside synthesis [13].

NPs are enzymes that catalyze the reversible phosphorolysis of nucleosides, typically using inorganic phosphate as a donor and generating a nucleobase and a sugar-1-phosphate as products. These enzymes play a crucial role in nucleotide metabolism by participating in the salvage pathways, which recycle nucleobases and nucleosides to form nucleotides [2]. Regarding their classification, structural analyses have revealed the presence of two distinct families of nucleoside phosphorylases, denoted as NP-I and NP-II [9,10]. Furthermore, based on substrate specificity, they are categorized into two classes: purine and pyrimidine nucleoside phosphorylases. Purine nucleoside phosphorylases (PNP, MTAP) selectively target purine nucleosides, including adenosine, guanosine, and 5′-deoxy-5′-methylthioadenosine, among others within the purine nucleoside spectrum. Conversely, pyrimidine nucleoside phosphorylases (PyNP, UP, TP) exhibit specificity towards pyrimidine nucleosides, such as uridine and thymidine [9,10].

**Figure 1:**
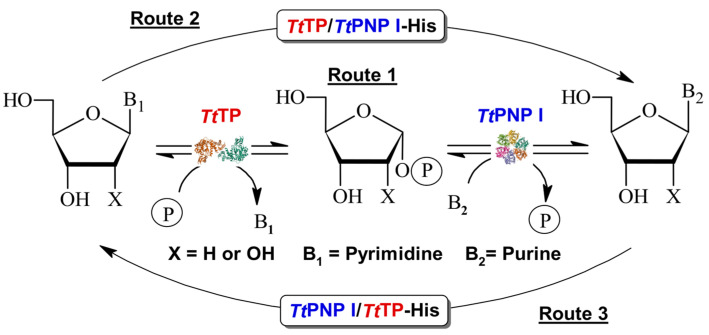
Transglycosylation reaction catalyzed by sequential action of TP and PNP I from *Thermus thermophilus*. Route 1: Cascade system using wt non-fused enzymes. Routes 2 and 3: One-pot synthesis using bifunctional fusion purine/pyrimidine NPs.

In recent years, nucleoside phosphorylases (NPs) have gained considerable interest as versatile catalysts for nucleoside APIs [4,9,10]. However, the prohibitive cost associated with various sugar-1-phosphate derivatives used as donors poses a significant challenge to their widespread industrial adoption [14,15]. Furthermore, while the thermodynamic equilibrium for purine nucleoside phosphorylases (PNPs) favors nucleoside synthesis, this is not the case for pyrimidine nucleoside phosphorylases (PyNP, UP, TP). To overcome this limitation, the most common approach for synthesizing nucleoside APIs involves the utilization of multi-enzymatic systems, such as PyNP/PNP, UP/PNP, or TP/PNP [16,17,18,19,20] (Figure 1, Route 1).

Nonetheless, a significant drawback of this methodology stems from the substantial costs associated with producing and purifying recombinant enzymes, necessitating the ability to reuse the catalyst for successive reactions to achieve cost-effectiveness. Furthermore, the integration of two or more enzymes within a multi-enzymatic system introduces operational complexities. These include challenges in achieving homogeneous catalysts when immobilizing the multi-enzyme system, as well as potential disparities in stability between the enzymes [18,21]. Considering these operational factors, an industrial standpoint favors the design of individual and homogeneous biocatalysts over heterogeneous enzymatic cascades whenever feasible.

Fusion proteins, also known as chimeric enzymes, are created by combining genetic material from two or more distinct enzymes to form a single functional unit. These engineered proteins retain the catalytic activities of their parent enzymes, often exhibiting novel or enhanced properties compared to the individual enzymes [22]. Genetic fusion holds the potential to amplify catalytic efficiency by bringing active sites into closer proximity, while also potentially enhancing gene expression, protein folding, and enzyme stability. In essence, fusion enzymes stand as a promising and versatile strategy within biocatalysis, offering avenues for the development of novel enzymatic processes endowed with heightened efficiency, specificity, and adaptability across diverse industrial and research realms [22,23,24,25]. Nonetheless, the meticulous design of fusion enzymes is imperative to ensure optimal performance.

Herein, we report the first detailed characterization of two engineered bifunctional enzymes, PNP I/TP-His-tagged (PNP I/TP-His) and TP/PNP I-His-tagged (TP/PNP I-His) (Figure 1, Routes 2 and 3), developed through the gene fusion of PNP I and TP from *Thermus thermophilus* [26,27,28,29,30]. Our experimental findings demonstrate that both engineered enzymes effectively catalyze the phosphorolytic cleavage of a broad spectrum of purine and pyrimidine ribo- (Guo, Ino, and Urd), and 2′-deoxyribonucleosides (dGuo, dIno, dUrd, and dThd). Comprehensive biochemical characterization, including the study of pH (6–8) and temperature (20–90 °C) effects, as well as long-term operational stability (60–80 °C), was conducted. Additionally, these fusion proteins demonstrated the ability to catalyze transglycosylation reactions between ribo- and 2′-deoxyribonucleosides and bases across a diverse range of purine and pyrimidine substrates. To further validate the practical application of these fused enzymes, we optimized the operational conditions (pH and temperature) for transglycosylation and successfully demonstrated the one-pot synthesis of various nucleoside analogs. This proof of concept underscores the potential of these novel fusion enzymes in biocatalytic applications.

## 2. Materials and Methods

### 2.1. Materials

The cell culture medium reagents were sourced from Difco (St. Louis, MO, USA). A triethyl ammonium acetate buffer was obtained from Sigma-Aldrich (Madrid, Spain). All additional reagents and organic solvents were procured from Symta (Madrid, Spain). Nucleosides and nucleobases utilized in this study were supplied by Biosynth Ltd. (Compton, UK).

### 2.2. Molecular Cloning

The codon-optimized genes for PNP I and TP from *Thermus thermophilus* HB27 (GenBank id: AE017221.1) were obtained as single constructs from GenScript Biotech (Piscataway, NJ, USA) in a pET22 vector. Subsequently, an overlap-extension PCR and golden gate assembly-based cloning approach was used to construct the fusion proteins [31]. We used a previously generated golden gate target vector based on pET28b (GG), in which backbone-located *Bsa*I sites were removed and a *Bsa*I entry site added in place of the multiple cloning site. The vector contained the coding region for a His-Tag on the C-terminus that was either left by cloning in the frame or removed by the introduction of a stop codon before the tag. Next, the TP and PNP I genes were amplified via PCR with extended primers that introduced appropriate overhangs for cloning and the molecular linker. A 12-amino-acid-long linker (SGGSGGSGGSAG), previously identified as optimal for another fusion construct [31], was introduced by incorporating half of the coding sequence into the reverse primer of the N-terminal fusion partner and the remaining half into the forward primer of the C-terminal partner.

For the construction of the two possible orientations of the His-tag-fused constructions, different PCRs were performed (Table 1). PCR success was confirmed by agarose gel electrophoresis and the corresponding products were subjected to a PCR clean up kit (Qiagen, Hilden, Germany). Next, an in-house-made golden gate reaction mix containing 15 U *Bsa*I, 15 U T4 ligase, and 1× T4 ligase buffer (New England Biolabs, Ipswich, MA, USA) was used to assemble 75 ng of the vector with a 3× molar excess of both inserts in the different reactions (Table 1). The assembly program ran in a thermocycler (Biorad, Utrecht, The Netherlands) by alternating 30× between 37 °C and 16 °C before a 20 min 55 °C enzyme deactivation step. Subsequently, 5 µL of this mix was used to transform competent *E. coli* NEB10 beta cells. A single colony was picked and verified by Sanger sequencing.

### 2.3. Enzyme Production and Purification

*E. coli* BL21 cells were transformed with the corresponding recombinant plasmid (pET28_TP/PNP I-His or pET28_PNP I/TP-His) and grown at 37 °C in a Luria Bertani medium supplemented with kanamycin at 50 μg/mL. Initially, protein overexpression was induced by adding 0.5 mM isopropyl-β-D-1-thiogalactopyranoside (IPTG) to the medium, followed by incubation at 20 °C for 5 h. Unfortunately, in these conditions, SDS-PAGE analysis revealed that almost no fusion protein was present in the soluble fraction. Consequently, different induction conditions were explored to enhance the solubility of the protein. These conditions involved the use of different concentrations of NaCl (0.2–0.8 M), glucose (1–2%), or IPTG (0.05–0.5 mM). Additionally, different cell growth conditions were also studied by extending the induction period to overnight at 20 °C (Figure S1).

Following the optimization of expression conditions, recombinant cells were harvested via centrifugation at 3600× *g*, and the resultant pellet was resuspended in 10 mM sodium phosphate buffer, pH 7.0. Crude extracts were prepared by cellular disruption using a digital sonicator, after which the lysates underwent centrifugation at 16,500× *g* for 30 min at 4 °C. Both fusion proteins, TP/PNP I-His and PNP I/TP-His, were purified using a standardized three-step procedure, which included an initial heat shock treatment, followed by an affinity chromatography step, and finally, a size exclusion chromatography. For this purpose, a partial purification was initiated by subjecting the clarified lysates to heat treatment at 80 °C for 30 min. Subsequently, the supernatant was centrifugated at 16,500× *g* for 30 min at 4 °C. The resulting clear lysate was then applied onto a 5 mL HisTrap FF column (GE Healthcare, Madrid, Spain) pre-equilibrated with a binding buffer (20 mM Tris-HCl buffer, pH 8.0, 100 mM NaCl, 10 mM imidazole). Bound proteins were eluted using a linear gradient of imidazole (10 to 500 mM). The resulting fractions containing the fusion proteins were pooled and concentrated, and subsequently purified by size exclusion chromatography using a HiLoad 16/60 Superdex 200 prep-grade column (GE Healthcare, Madrid, Spain) pre-equilibrated in 50 mM phosphate buffer, pH 7.0. Finally, fractions containing the enzyme were identified, pooled, and dialyzed against 10 mM phosphate buffer, pH 7.0. Once dialyzed, enzymes were concentrated, and the purity of samples was evaluated by SDS-PAGE. Protein concentration was determined using UV_280_ nm spectroscopy, utilizing a molar extinction coefficient of ε_280_ = 62,340 M^−1^ cm^−1^, calculated from the amino acid sequence of the protein.

To facilitate a comparative analysis of phosphorolysis activity results between fusion proteins and their non-fused counterparts (PNP I and TP from *Thermus thermophilus* HB27), both *Tt*PNP I (UniProtKB ID: Q72IR2) and *Tt*TP (UniProtKB ID: Q5SHF9) were produced and purified for subsequent experiments. The procedures for expression and purification were conducted following previously described methods [26,27,28,29,30].

### 2.4. Standard Assay for Enzyme-Mediated Phosphorolysis of Nucleosides

The experimental procedure for assessing the phosphorylase activity of the soluble enzymes was adapted from previously established methodologies as described in the literature [26,27,28,29,30]. Specifically, the enzyme-mediated phosphorylase assay was conducted by incubating 0.3 µg of pure enzyme with 5 mM (2′-deoxy)ribonucleoside in 50 mM sodium phosphate buffer, pH 7.0, at 60 °C and 300 rpm for 10 min. Subsequently, the enzymatic reaction was terminated by adding 40 μL of cold methanol in an ice bath, followed by heating for 5 min at 95 °C. Upon centrifugation at 8500× *g* for 5 min, the resulting samples were half-diluted with water, and the presence of the released nucleobase was analyzed and quantified through HPLC. All measurements were performed in triplicate with a maximum error below 5%.

For accurate comparisons between the enzymatic activities of the fusion enzymes and their native counterparts, only the mass of the fusion enzyme corresponding to the specific domain of interest was considered in the activity calculations. This method ensures that the activity measured is directly attributable to the relevant domain within the fusion enzyme. Detailed procedures for this calculation are provided in the Supplemental Material section.

### 2.5. Biochemical Characterization

To establish the optimal operational conditions, the influence of pH and temperature on enzyme activity was systematically evaluated. In this context, the pH-dependent activity of both the TP and PNP I domains was tested for TP/PNP I-His and PNP I/TP-His at different pH values using a 50 mM sodium phosphate buffer over a pH range of 6–8. Similarly, the effect of temperature on the phosphorolytic activity for both TP and PNP I domains was assayed across the 20–90 °C interval in both fusion enzymes.

Additionally, the effect of temperature on enzyme stability was investigated by incubating 0.3 µg of the purified enzyme in 10 mM sodium phosphate buffer (pH 7.0) for 48 h at various temperatures (60–80 °C). Throughout these incubation periods, phosphorylase activity was systematically assayed over 2′-deoxyinosine (dIno) and 2′-deoxyuridine (dUrd) under standard assay conditions previously described. The thermal stability of both fusion proteins was defined as the relative activity between the first and successive reactions.

### 2.6. Standard Assay for Transglycosylation Activity

To evaluate the transglycosylase activity of the fusion enzymes, a series of transglycosylation reactions were performed using a diverse panel of nucleoside donors and nucleobase acceptors. For this purpose, 1 µg of the pure enzyme was added to a reaction mixture containing 1 mM (2′-deoxy)ribonucleoside and 1 mM base in 50 mM sodium phosphate buffer, pH 7. The reaction proceeded at 65 °C with agitation at 300 rpm for 30 min in a final volume of 40 µL. Subsequently, the enzymatic reaction was quenched, and reaction products were processed and analyzed as described previously for the phosphorylase assay. All measurements were performed in triplicate with a maximum error below 5%.

### 2.7. Influence of pH and Temperature on Transglycosylation Capability of TP/PNP I-His and PNP I/TP-His

To optimize the operational conditions for future synthetic applications, the effects of pH and temperature on the transglycosylase activity of both fusion enzymes were assessed using the same experimental procedures as those applied for the phosphorylase reaction (see Section 2.4). Given that the fusion proteins contain both purine and pyrimidine phosphorylase domains, two different reactions, dIno + Thy and dThd + Hyp, were selected as models to evaluate the pH and temperature dependence of the transglycosylation activity of TP/PNP I-His and PNP I/TP-His.

### 2.8. Enzymatic Synthesis of Nucleoside Analogs

To evaluate the potential use of TP/PNP I-His and PNP I/TP-His as catalysts, the enzymatic production of various ribo- and 2′-deoxyribonucleoside analogs was achieved through different transglycosylation reactions. To this end, a comprehensive screening was conducted, employing various natural (2′-deoxy)ribonucleosides as donors and a selection of nucleobase derivatives as acceptors. The experimental procedure involved the incubation of 1 µg of the pure enzyme with 1 mM nucleoside donor and 1 mM of the corresponding acceptor nucleobase in 50 mM sodium phosphate buffer, pH 7, in a final volume of 40 μL. The reaction mixtures were incubated at 65 °C and 300 rpm in an orbital shaker for 24 h. Afterward, the enzymatic reaction was stopped, and the resulting products were processed and analyzed as previously described. All experiments were conducted in triplicate, ensuring a maximum error margin below 5%.

### 2.9. Analytical Methods

The identification and quantification of products were performed by HPLC analysis using an ACE 5 μm C18-PFP 250 mm × 46 mm column under the following conditions: (i) a continuous gradient (100–90% 0.1 M triethylammonium acetate and 0–10% acetonitrile) for 15 min and (ii) an isocratic elution (90% 0.1 M triethylammonium acetate and 10% acetonitrile) for 7 min. The flow rate was fixed at 0.9 mL/min (180 bar pressure) and the UV detector was set at 254, 250, 240, and 230 nm. Commercial nucleosides and bases were used as HPLC standards to confirm the reaction products.

Retention times for the reference nucleobases and nucleosides, hereafter abbreviated following the recommendations of the IUPAC-IUB Commission on Biochemical Nomenclature, were as follows: guanine (Gua), 7.5 min; hypoxanthine (Hyp), 7.2 min; uracil (Ura), 5.6 min; thymine (Thy), 10 min; 5-fluorouracil (5-FUra), 5.4 min; 5-bromouracil (5-BrUra), 11.5 min; 5-chlorouracil (5-ClUra), 9 min; 5-iodouracil (5-IUra), 13 min; 6-methylpurine (6-MetPur), 12.3 min; 6-chloropurine (6-ClPur), 15.0 min; 6-mercaptopurine (6-MP), 10.0 min; guanosine (Guo), 10.9 min; 2′-deoxyguanosine (dGuo), 12.1 min; inosine (Ino), 10.7 min; 2′-deoxyinosine (dIno), 12.4 min; uridine (Urd), 8.7 min; 2′-deoxyuridine (dUrd), 9.8 min; thymidine riboside (Thd), 13.5 min; 2′-deoxythimidine (dThd), 14.3 min; 5-fluorouridine (5-FUrd), 7.9 min; 5-fluoro-2′-deoxyuridine (5-FdUrd), 8.8 min; 5-chlorouridine (5-ClUrd), 13.2 min; 5-chloro-2′-deoxyuridine (5-CldUrd), 13.9 min; 5-bromouridine (5-BrUrd), 15.2 min; 5-bromo-2′-deoxyuridine (5-BrdUrd), 16.5 min; 5-iodouridine (5-IUrd), 17.9 min; 5-iodo-2′-deoxyuridine (5-IdUrd), 20.1 min; 6-methylpurine ribose (6-MetPur Rib), 20.1 min; 6-methylpurine-2′-deoxyribose (6-MetPur dRib), 27.1 min; 6-chloropurine ribose (6-ClPur Rib), 17.6 min; 6-chloropurine-2′-deoxyribose (6-ClPur dRib), 24.2 min; 6-mercaptopurine ribose (6-MP Rib), 12.9 min; 6-mercaptopurine-2′-deoxyribose (6-MP dRib), 14.2 min.

## 3. Results and Discussion

### 3.1. Engineering, Production, and Purification of Bifunctional Fusion Enzymes

**Figure 2:**
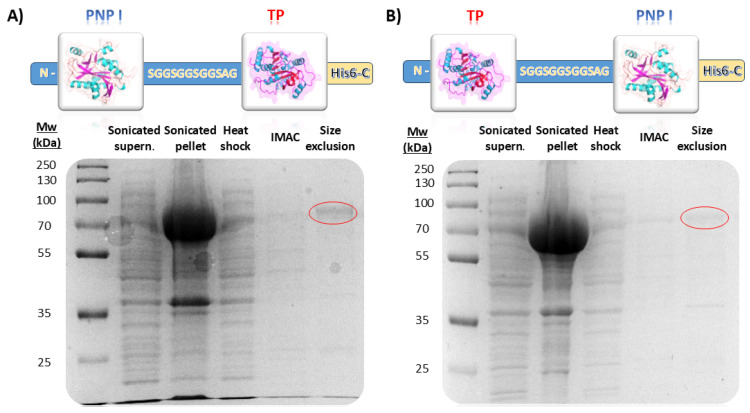
Evaluation of different purification steps for (**A**) PNP I/TP-His and (**B**) TP/PNP I-His. IMAC (Immobilized Metal Affinity Chromatography).

In this study, we successfully engineered a bifunctional fusion enzyme combining a type I purine nucleoside phosphorylase (PNP I) and a thymidine phosphorylase (TP) from *Thermus thermophilus*. The fusion constructs, designated as pET28_TP/PNP I-His and pET28_PNP I/TP-His, included a flexible 12-amino-acid-long linker (SGGSGGSGGSAG) between the two domains to facilitate proper domain spacing and folding (Figure 2). TP/PNP I-His and PNP I/TP-His were expressed in *Escherichia coli* BL21(D3) and subsequently purified according to a three-step protocol, including a heat shock treatment, affinity chromatography, and finally, size exclusion chromatography (Figure 2). The SDS-PAGE analysis confirmed the expected molecular weight of the fusion protein, approximately 74 kDa consistent with the molecular mass calculated from the amino acid sequence (74.52 kDa), indicating successful expression and purification. Regarding the unfused proteins, both *Tt*PNP I and *Tt*TP were effectively expressed in *E. coli* BL21(D3) and subsequently purified following the same protocol (Figure S2).

Both fusion proteins, TP/PNP I-His and PNP I/TP-His, demonstrated solubility at room temperature; however, when stored at 4 °C, their solubility was reduced, leading to the formation of insoluble aggregates. Consequently, we chose 25 °C as the optimal storage temperature for both proteins. Additionally, analytical ultracentrifugation studies were performed to elucidate the oligomeric state of the proteins in the solution. Unfortunately, experimental data revealed that both proteins generate a range of oligomeric species in solution. This finding is consistent with our expectations, given the known structural properties of the proteins. Specifically, the wild-type *Tt*PNP I is known to form a homohexamer, which aligns with the observed complexity of oligomeric species. Conversely, *Tt*TP is reported to exist as a homodimer, which also correlates with the presence of distinct oligomeric forms. This behavior reflects the fundamental differences in the quaternary structures of these proteins, as supported by previous studies [26,27,28,29,30]. To address the challenge of the formation of multiple oligomeric species in solution, several approaches were considered. These included varying the concentration of salts and adjusting the pH to optimize the protein’s environment and stabilize a more homogeneous oligomeric form. Additionally, the application of cryoprotectants such as glycerol, along with other stabilizing agents like detergents, was investigated to reduce protein aggregation and enhance stability. Despite these efforts, the experimental problem persisted, hindering the biophysical characterization of the enzyme.

### 3.2. Biochemical Characterization of the Phosphorolytic Activity in the Fusion Enzymes

To optimize synthetic applications, it is crucial to investigate the substrate specificity of the fusion enzymes and determine the conditions for achieving maximum activity. Initially, we assessed the phosphorolytic activity of PNP I/TP-His and TP/PNP I-His across a diverse array of purine and pyrimidine (2′-deoxy)ribonucleosides (Table 2).

As demonstrated in Table 2, the phosphorolytic activity on 6-oxopurine nucleosides for both fusion enzyme TP/PNP I-His is consistent with the activity observed in the non-fused counterpart (*Tt*PNP I), with retained activity values ranging from 60% to 100%. Interestingly, a slight increase in enzymatic activity, compared to the non-fused enzymes, was observed when 6-oxopurine 2′-deoxynucleosides were used as substrates. However, the phosphorolytic activity on pyrimidine nucleosides is significantly compromised in the fusion enzymes, with retained activity values below 38% in all cases. These findings suggest that the oligomeric state of the fusion enzymes exerts a more pronounced impact on the TP domain than on the PNP I domain. Given that the wild-type *Tt*PNP I exists as a hexamer and the wild-type *Tt*TP as a dimer, it is likely that the PNP I domain in the fusion enzymes retains a high oligomeric state. This high oligomeric assembly of the PNP I domain may impose steric hindrance or induce structural constraints that interfere with the TP domain’s ability to adopt its optimal dimeric configuration, thereby impairing its catalytic efficiency. This differential impact on the TP domain highlights the challenges associated with maintaining functional integrity in fusion proteins where the oligomeric states of the constituent domains are dissimilar. Moreover, wild-type *Tt*TP exhibits inhibition by nucleobases at low concentrations [29]. This effect may be further amplified in the fusion enzymes due to the aforementioned factors.

The effects of temperature and pH on the phosphorolytic activity of each domain in both PNP I/TP-His and TP/PNP I-His fusion enzymes were assessed (Figures S3 and S4). Temperature profiles revealed that dIno phosphorolysis (activity assay reflecting PNP I domain activity) exhibited high activity within the 60–90 °C range in both fusion enzymes. In contrast, dUrd phosphorolysis (activity assay reflecting TP domain activity) showed activities of ≥60% within the narrower 80–90 °C range. Additionally, pH profiles indicated that dIno phosphorolysis (PNP I domain) maintained high activity (≥80%) within a pH range of 6 to 7.5 across both fusion enzymes, whereas this level of activity for dUrd phosphorolysis (TP domain) was restricted to a pH range of 6 to 6.5. These results are consistent with those described for the non-fused enzymes, *Tt*PNP I and *Tt*TP, which display similar operational features [26,28,29].

**Figure 3:**
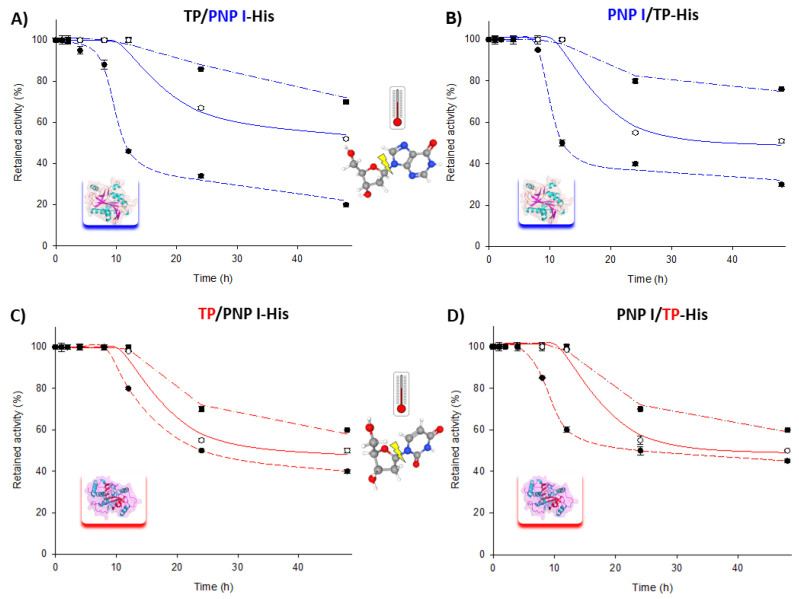
Time course of thermal inactivation of PNP I/TP-His and TP/PNP I-His at (■) 60 °C, (○) 70 °C, and (●) 80 °C in 10 mM sodium phosphate, pH 7.0. Thermal stability of PNP I domain (highlighted in blue) in (**A**) TP/PNP I-His and (**B**) PNP I/TP-His. Thermal stability of TP domain (highlighted in red) in (**C**) TP/PNP I-His and (**D**) PNP I/TP-His.

Moreover, the long-term stability of the fusion enzymes was assessed over a 60–80 °C range for extended periods. As shown in Figure 3, both fusion enzymes retained approximately 70–80% of their phosphorolytic activity for both the TP and PNP I domains after 48 h at 60 °C. Similarly, at 70 and 80 °C, the trend was consistent for both fusion enzymes, with a t_1/2_ of approximately 24–48 h at 70 °C and less than 10 h at 80 °C for the phosphorolytic activity of the PNP I domain. Remarkably, the TP domain exhibited a similar t_1/2_ of around 24 h at both 70 and 80 °C.

These results are particularly noteworthy given the inherent challenges in nucleoside phosphorylase-catalyzed processes, where the poor solubility of many nucleosides and nucleobases in aqueous solution typically necessitates harsh reaction conditions (e.g., basic/acid environments, heat, and cosolvents) to achieve effective substrate loading beyond the low millimolar range. The demonstrated long-term stability and high phosphorolytic activity of our fusion enzymes at elevated temperatures (60–80 °C) underscore their potential to operate efficiently under these demanding conditions, thereby offering a robust solution to the solubility limitations commonly encountered in such processes.

### 3.3. Biochemical Characterization of the Transglycosylation Capability of PNP I/TP-His and TP/PNP I-His Fusion Enzymes

The reversible nature of the NP-catalyzed reactions (transglycosylation) has already been exploited for synthesizing nucleosides through the combination of an acceptor nucleobase and a pentose-1P. However, the high price of pentose-1P derivatives is a serious constraint for their industrial application as catalysts for enzymatic synthesis of modified nucleosides. Moreover, while the thermodynamic equilibrium for purine nucleoside phosphorylases (PNPs) favors nucleoside synthesis, this is not true for pyrimidine nucleoside phosphorylases (PyNP, UP, TP). To overcome this limitation, the most common approach for synthesizing nucleoside analogs involves using multi-enzymatic systems, such as PyNP/PNP, UP/PNP, or TP/PNP [16,17,18,19,20].

In this context, following the biochemical characterization of the phosphorolytic activity of the fusion enzymes, we evaluated the transglycosylation capability of both fusion systems. To achieve this, a general screening was first conducted, focusing on the enzymatic synthesis of ribo- and 2′-deoxyribonucleosides from the combination of pyrimidine/purine (2′-deoxy)ribonucleosides and purine/pyrimidine bases. As shown in Table 3, the highest conversion rates were achieved when the donor and acceptor nucleosides were of the same type—either both purines or both pyrimidines—indicating that only a single domain of the fusion enzyme was actively involved in the catalysis. Notably, the transglycosylation reaction using dUrd, Urd, or dThd as donors and Hyp as an acceptor exhibited remarkable activity. However, this trend was not observed in other transglycosylation reactions between a purine nucleoside and a pyrimidine nucleobase, which displayed conversions below 5%.

**Figure 4:**
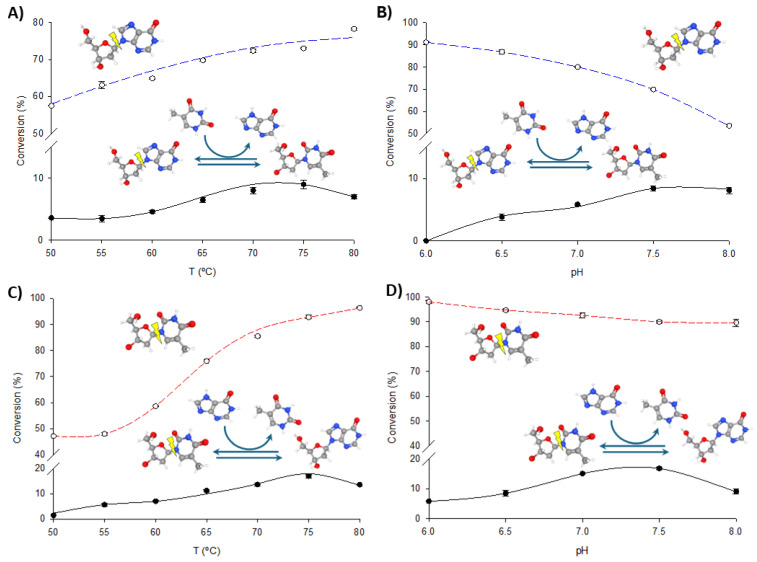
Biochemical characterization of TP/PNP I-His. (**A**) Effect of temperature on phosphorolysis (dIno, blue dotted line) and transglycosylation (dIno + Thy, dark line). (**B**) Effect of pH on phosphorolysis (dIno, blue dotted line) and transglycosylation (dIno + Thy, dark line). (**C**) Effect of temperature on phosphorolysis (dThd, red dotted line) and transglycosylation (dThd + Hyp, dark line). (**D**) Effect of pH on phosphorolysis (dThd, red dotted line) and transglycosylation (dThd + Hyp, dark line).

**Figure 5:**
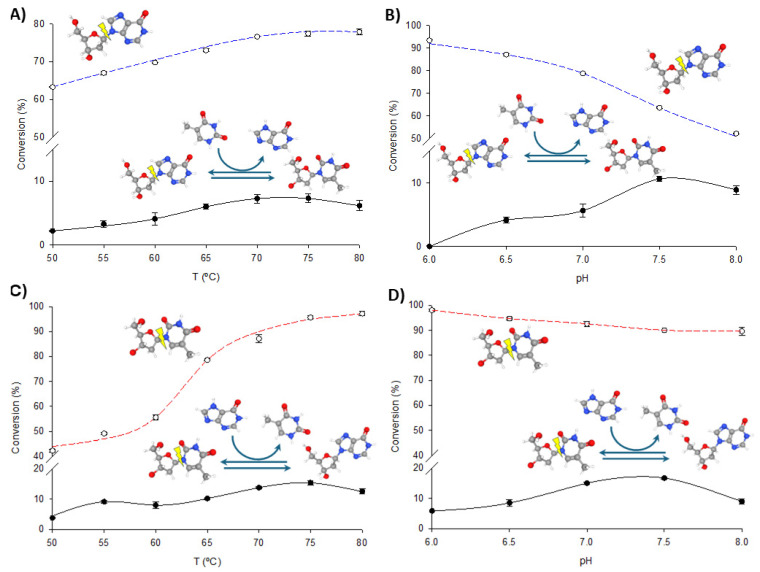
Biochemical characterization of PNP I/TP-His. (**A**) Effect of temperature on phosphorolysis (dIno, blue dotted line) and transglycosylation (dIno + Thy, dark line). (**B**) Effect of pH on phosphorolysis (dIno, blue dotted line) and transglycosylation (dIno + Thy, dark line). (**C**) Effect of temperature on phosphorolysis (dThd, red dotted line) and transglycosylation (dThd + Hyp, dark line). (**D**) Effect of pH on phosphorolysis (dThd, red dotted line) and transglycosylation (dThd + Hyp, dark line).

To optimize the operational conditions for further synthetic applications, the effects of pH and temperature on the transglycosylation and phosphorolytic activity of fused enzymes were studied using dIno + Thy and dThd + Hyp as model reactions. As illustrated in Figure 4 and Figure 5, increasing the temperature enhances both phosphorolysis and transglycosylation activities of the fusion enzymes, consistent with their thermophilic nature. The highest transglycosylation conversion is achieved in the 65–80 °C range. However, the effect of pH on these activities shows a distinct pattern. Maximum phosphorolytic activity is observed at low to neutral pH (6.0–6.5), decreasing at higher pH levels. In contrast, transglycosylation activity is minimal at pH 6.0 but peaks at higher pH values (7.0–8.0).

Despite these observations, experimental data consistently demonstrate that phosphorolysis activity exceeds transglycosylation activity under all conditions, regardless of the substrate type (purine or pyrimidine nucleosides) or the enzyme variant (TP/PNP I-His or PNP I/TP-His). One potential explanation for this phenomenon is the substrate channeling effect, in which the intermediate is directly transferred from one enzyme’s active site to the next, bypassing random diffusion and thereby accelerating the reaction [22,32,33,34,35]. This effect depends on the close spatial proximity between enzyme domains but is most beneficial when intermediate concentrations are low. In our case, the significantly higher phosphorolysis activity compared to transglycosylation suggests that the conditions may not be conducive to effective channeling. As shown in Figure 4 and Figure 5, the pH profiles reveal that phosphorolytic activity decreases at higher pH values, whereas transglycosylation activity increases. This observation supports the substrate channeling hypothesis and suggests that effective channeling is less advantageous under conditions that favor higher phosphorolytic activity.

Additionally, the oligomeric distribution of TP/PNP I-His and PNP I/TP-His in solution may contribute to the reduced transglycosylation activity. The PNP I domain, which forms a hexamer in its native state, and the TP domain, which is a dimer, generate a variety of oligomeric species upon fusion. The spatial organization of multi-enzyme complexes is essential for overcoming barriers between different enzyme classes, avoiding mutual inhibition, limiting the long-range diffusion of intermediates, and enhancing reaction efficiency. Inappropriate oligomeric assembly in these fusion enzymes may disrupt this organization, further diminishing the efficiency of transglycosylation reactions, which require precise alignment and coordination between the PNP I and TP domains. These issues, including misfolding and improper assembly, are well documented in the literature [22,32,33]. To address these challenges, future work should explore alternative linker designs, such as flexible versus rigid linkers, varying lengths, and different orientations, to optimize the fusion enzyme system [36,37,38].

### 3.4. Enzymatic Synthesis of Nucleoside Analogs Using PNP I/TP-His and TP/PNP I-His Fusion Enzymes

The development of fusion enzymes in our study is specifically aimed at overcoming the limitations associated with the use of nucleoside phosphorylases (NPs) in nucleoside API synthesis. Traditional NP-based methods, while effective, face significant challenges such as the high cost of sugar-1-phosphate donors and the need to manage various types of NPs with differing catalytic properties [4,9,10,14,15]. Although multi-enzymatic systems combining purine and pyrimidine NPs have been explored as a solution [16,17,18,19,20,39,40], their practical application remains constrained by the costs of enzyme production and purification, and the difficulty of maintaining consistent catalytic performance across batches [18,21]. Our approach addresses these challenges by creating bifunctional fusion enzymes, which combine the activities of purine nucleoside phosphorylase (PNP) and thymidine phosphorylase (TP) in a single protein. This fusion reduces the complexity of enzyme production and eliminates the need for separate purification processes for each enzyme, significantly lowering the overall cost. Additionally, fusion enzymes offer the advantage of ensuring consistent stoichiometry and catalytic performance, circumventing the heterogeneity often observed in co-immobilized multi-enzyme systems [41,42,43]. By consolidating multiple enzymatic activities into a single protein, our engineered fusion enzymes also provide a more stable and uniform biocatalyst, which is crucial for industrial applications. This approach simplifies the optimization of reaction conditions, as both catalytic domains operate under the same set of environmental parameters, further enhancing efficiency. Thus, the development of these bifunctional fusion enzymes represents a significant advancement in addressing the existing limitations of NP-based multi-enzymatic systems.

While limited progress has been made in addressing these issues, a notable exception is the work by Liu and colleagues, who engineered a promiscuous nucleoside phosphorylase with both PNP and uridine phosphorylase (UP) activities using an insertion–deletion strategy [44]. Although their approach is promising, our study provides a different solution by designing fusion enzymes that retain the specific activities of PNP and TP, tailored for one-pot, single-step synthesis reactions. This fusion strategy simplifies the optimization of reaction conditions, ensuring that both catalytic domains function efficiently under the same conditions, making it particularly suitable for industrial applications.

To showcase the potential of the PNP I/TP-His and TP/PNP I-His fusion enzymes, we performed the one-pot synthesis of various purine and pyrimidine ribo- and 2′-deoxyribonucleoside analogs. This proof of concept was achieved through straightforward transglycosylation reactions, conducted under mild and sustainable conditions. As shown in Table 4, different nucleoside donors (dUrd, Urd, and Guo) and nucleobase acceptors (6-mercaptopurine, 6-MP; 6-methylpurine, 6-MetPur; 6-chloropurine, 6-ClPur; 5-fluorouracil, 5-FUra; 5-iodouracil, 5-IUra; 5-chlorouracil, 5-ClUra; 5-bromouracil, 5-BrUra) were assayed.

## 4. Conclusions

Herein, we present, for the first time, the design and biochemical characterization of two fusion enzymes, PNP I/TP-His and TP/PNP I-His, which combine purine nucleoside phosphorylase (PNP) and thymidine phosphorylase (TP) activities. Our results demonstrate that while the PNP I domain retains its full phosphorolytic activity compared to the non-fused *Tt*PNP I, the TP domain’s phosphorolytic activity is significantly reduced in the fusion construct. Thermal stability studies revealed that both enzyme domains maintain high activity over extended periods at elevated temperatures (60–80 °C).

Notably, these fusion enzymes are capable of catalyzing transglycosylation reactions between purine nucleoside donors and pyrimidine base acceptors, and vice versa. However, phosphorolysis consistently outperformed transglycosylation under all tested conditions. The balance between these activities is modulated by both pH and temperature: phosphorolysis is most active at low to neutral pH, whereas transglycosylation peaks at higher pH levels. The oligomeric mismatch between the PNP I and TP domains, with PNP I typically forming hexamers and TP existing as dimers, likely contributes to the presence of diverse oligomeric species in solution, which does not allow the optimal alignment of active sites, resulting in reduced efficiency for transglycosylation reactions. As a proof of concept, we successfully demonstrated the synthesis of various purine and pyrimidine ribo- and 2′-deoxyribonucleoside analogs using one-pot transglycosylation reactions catalyzed by the fusion enzymes PNP I/TP-His and TP/PNP I-His. These findings establish a fundamental basis for advancing the development of fusion enzymes as multifunctional biocatalysts in nucleoside analog synthesis, thereby enhancing the potential for more efficient and sustainable processes in industrial applications. Future studies should explore alternative linker designs and orientations within the fusion constructs to enhance spatial organization and improve the functional synergy of the enzyme domains.

## Figures and Tables

**Table 1:** Molecular cloning, PCR reactions, and primer sequences. *Bsa*I sites are underlined.

Construct	Backbone	PCR 5′ Construct		PCR 3′ Construct	
		FW Primer	RV Primer	FW Primer	RV Primer
pET28_TP/PNP I-His	pET28-GG-His	GAGGTCTCGTGGTATGAACCCGGTGGTTTTCATC	GAGGTCTCAGAACCACCCGATCCACCACTAATCGCCTCCAGCACCAG	GAGGTCTCAGTTCAGGAGGAAGTGCGGGTATGAGCCCGATTCATGTGC	GTGGTCTCGCAAGCACTTCCAGCACCGCTTC
pET28_PNP I/TP-His	pET28-GG-His	GAGGTCTCGTGGTATGAGCCCGATTCATGTGC	GAGGTCTCAGAACCACCCGATCCACCACTCACTTCCAGCACCGCTTC	GAGGTCTCAGTTCAGGAGGAAGTGCGGGTATGAACCCGGTGGTTTTCATC	GTGGTCTCGCAAGAATCGCCTCCAGCACCAG

**Table 2:** Phosphorolytic activity of PNP I/TP-His and TP/PNP I-His.

	Specific Activity (U/mg_enz_) ^a^			
Substrates	TP/PNP I-His ^b^	PNP I/TP-His ^b^	*Tt*PNP I	*Tt*TP
**Ado**	n.d.	n.d.	n.d.	n.d.
**Guo**	6.4 ± 0.1	7.5 ± 0.1	8.8 ± 0.1	n.d.
**Ino**	8.1 ± 0.1	8.6 ± 0.1	14.3 ± 0.9	n.d.
**Cyd**	n.d.	n.d.	n.d.	n.d.
**Urd**	5.3 ± 0.2	3.0 ± 0.1	n.d.	16.2 ± 0.5
**dAdo**	n.d.	n.d.	n.d.	n.d.
**dGuo**	12.5 ± 0.1	12.5 ± 0.3	8.6 ± <0.1	n.d.
**dIno**	26.7 ± 0.1	26.9 ± 0.2	22.1 ± 1.0	n.d.
**dCyd**	n.d.	n.d.	n.d.	n.d.
**dUrd**	13.4 ± 0.2	8.6 ± 0.1	n.d.	34.2 ± 1.8
**dThd**	10.1 ± <0.1	7.2 ± 0.1	n.d.	32.2 ± 2.0

^a^ Reaction conditions: 0.3 μg of pure enzyme in 40 μL at 60 °C, 10 min. [Substrates] = 1 mM, 50 mM sodium phosphate buffer, pH 7.0. ^b^ Normalized activity of the fusion enzymes adjusts for contribution of each enzymatic domain within fusion construct. n.d. not detected.

**Table 3:** Enzymatic production of natural nucleosides by transglycosylation catalyzed by PNP I/TP-His and TP/PNP I-His.

		Percentage of Conversion (%)						
		PNP I/TP-His ^a^				TP/PNP I-His ^a^		
	Acceptor	Hyp	Ura	Thy		Hyp	Ura	Thy
Donor								
**Ino**		-	3 ± 0.3	2 ± 0.3			3 ± 0.3	2 ± 0.3
**dIno**		-	4 ± 0.1	7 ± 0.5			3 ± 0.3	6 ± 0.3
**Guo**		24 ± 0.3	3 ± 0.1	-		22 ± 1.3	3 ± 0.7	-
**dGuo**		26 ± 1.0	3 ± 0.4	-		26 ± 1.9	3 ± 0.8	-
**Urd**		31 ± 0.8	-	29 ± 0.8		31 ± 0.3	-	29 ± 0.9
**dUrd**		12 ± 0.9	-	17 ± 0.3		19 ± 0.2	-	17 ± 1.0
**dThd**		10 ± 0.9	-	-		11 ± 0.3	-	-

^a^ Exp. conditions: 0.3 μg enz, 65 °C, 30 min. [Substrates] = 1 mM, 50 mM sodium phosphate, pH 7.0.

**Table 4:** Enzymatic production of nucleoside analogs by transglycosylation catalyzed by PNP I/TP-His and TP/PNP I-His.

		Percentage of Conversion (%)						
		PNP I/TP-His ^a^				TP/PNP I-His ^a^		
	Donor	dUrd	Urd	Guo		dUrd	Urd	Guo
Acceptor								
**6-MP**		n.d.	14 ± 0.3	-		n.d.	4 ± 0.3	-
**6-MetPur**		28 ± 1.5	7 ± 0.3	-		13 ± 0.6	2 ± 0.3	-
**6-ClPur**		11 ± 0.8	4 ± 0.9	-		22 ± 1.3	3 ± 0.3	-
**5-FUra**		-	-	10 ± 0.8		-	-	11 ± 0.7
**5-IUra**		-	-	5 ± 0.3		-	-	5 ± 0.7
**5-ClUra**		-	-	4 ± 0.3		-	-	n.d.
**5-BrUra**		-	-	n.d.		-	-	n.d.

^a^ Exp. conditions: 3 μg enz, 65 °C, 24 h. [Substrates] = 1 mM, 50 mM sodium phosphate, pH 7.0. n.d. not detected.

## Data Availability

All data related to this manuscript are available upon request.

## Supplementary Materials

The following supporting information can be downloaded at: https://www.mdpi.com/article/10.3390/biom14091196/s1, Figure S1: Optimization of protein overexpression using different concentrations of NaCl (0.2 M to 0.8 M), glucose (1% to 2%), or IPTG (0.05 mM to 0.5 mM). Figure S2: Evaluation of different purification steps for *Tt*PNP I and *Tt*TP. Lane 1: Markers. Lanes 2–4: Purification process for *Tt*PNP I. Lanes 6–8: Purification process for *Tt*TP. Figure S3: Effect of T on both PNP I domain (2′-deoxyinosine phosphorolysis, blue dotted line) and TP domain (2′-deoxyuridine phosphorolysis, red dotted line) in TP/PNP I-His and PNP I/TP-His fusion enzymes. Figure S4: Effect of pH on both PNP I domain (2′-deoxyinosine phosphorolysis, blue dotted line) and TP domain (2′-deoxyuridine phosphorolysis, red dotted line) in TP/PNP I-His and PNP I/TP-His fusion enzymes.
