# Supplementary material for: Engineering a Bifunctional Fusion Purine/Pyrimidine Nucleoside Phosphorylase for the Production of Nucleoside Analogs
Source: Biomolecules. 2024 Sep 23;14(9):1196. doi: 10.3390/biom14091196 (PMC11430618; doi:10.3390/biom14091196)
Supplement: Supplementary file 1 [file biomolecules-14-01196-s001.zip › biomolecules-3205287-supplementary.pdf]

biomolecules

MDPI

Article

Engineering a Bifunctional Fusion Purine/Pyrimidine Nucleoside Phosphorylase for the Production of Nucleoside Analogs

Daniel Hormigo [1,†], Jon Del Arco [1,†], Javier Acosta [1], Maximilian J. L. J. Fürst [2] and Jesús Fernández-Lucas [1,3,4,*]

1 Applied Biotechnology Group, Universidad Europea de Madrid, Urbanización El Bosque, Villaviciosa de Odón, 28670 Madrid, Spain; daniel.hormigo@universidadeuropea.es (D.H.); jon.delarco@universidadeuropea.es (J.D.A.); javier.acosta@universidadeuropea.es (J.A.)
2 Molecular Enzymology Group, University of Groningen, Feringa Building, 9747 AG Groningen, The Netherlands
3 Grupo de Investigación en Ciencias Naturales y Exactas, GICNEX, Universidad de la Costa, CUC, Calle 58 #55-66, Barranquilla 080002, Colombia
4 Department of Biochemistry and Molecular Biology, Faculty of Biology, Universidad Complutense de Madrid, C. de José Antonio Novais, 12, 28040 Madrid, Spain
* Correspondence: jesusf08@ucm.es
† These authors contributed equally to this work.

Supplemental material

1. Materials and methods

1.1. Amino acid sequences

The linker sequence between enzymes is highlighted in pink, while the linker sequence between the enzyme and the His tag (HHHHHH), determined by the pET plasmid system, is highlighted in red.

PNP I/TP- His

MSPIHVRAHPGDVAERVLLPGDPGRAEWIAKTFLQNPRRYNDHRGLWGYTGLYK
GVPVSVQTTGMGTPSAAIVVEELVRLGARVLVRVGTAGAASSDLAPGELIVAQGA
VPLDGTTRQYLEGRPYAPVPDPEVFRALWRRAEALGYPHRVGLVASEDAFYATTPE
EARAWARYGVLAFEMEASALFLLGRMRGVRTGAILAVSNRIGDPELAPPEVLQEG
VRRMVEVALEAVLEVSGGSGGSGGSAGMNPVVFIREKREGKKHRREDLEAFLLGY
LRDEVPDYQVAAWLMAAFLRGLDAEETLWLTETMARSGKVLDLSGLPHPVDKHS
SGGVGDKVSLVVGPILAASGCTFAKMSGRGLAHTGGTIDKLESVPGWRGEMTEAE
FLERARRVGLVIAAQSPDLAPLDGKLYALRDVTATVESVPLIASSIMSKKLAAGARSI
VLDVKVGRGAFMKTLEEARLLAKTMVAIGQGAGRRVRALLTSMEAPLGRAVGNA
IEVREAIGALKGEGPEDLLEVALALAEEALKLEGLDPALARKALEGGAALEKFRAF
LEAQGGDPRAVEDFSLLPLAEEHPLRAEREGVVQEVDAYKVGLAVLALGGGRKRK
GEPIDHGVGVYLLKKPGDRVERGEALALVYHRRRGLEEALGHLREAYALGEEAHP
APLVLEAILETSLGIYSAQSASGDAANLVEHHHHHH

Mw: 74,517.44 Da **ε280 = 62,340 M^{-1} cm^{-1}**

TP/PNP I-His

MNPVVFIREKREGKKHRREDLEAFLLGYLRDEVPDYQVAAWLMAAFLRGLDAEE
TLWLTETMARSGKVLDLSGLPHPVDKHSSGGVGDKVSLVVGPILAASGCTFAKMS
GRGLAHTGGTIDKLESVPGWRGEMTEAEFLERARRVGLVIAAQSPDLAPLDGKLY
ALRDVTATVESVPLIASSIMSKKLAAGARSIVLDVKVGRGAFMKTLEEARLLAKTM
VAIGQGAGRRVRALLTSMEAPLGRAVGNAIEVREAIGALKGEGPEDLLEVALALA
EEALKLEGLDPALARKALEGGAALEKFRAFLEAQGGDPRAVEDFSLLPLAEEHPLR
AEREGVVQEVDAYKVGLAVLALGGGRKRKGEPIDHGVGVYLLKKPGDRVERGEA
LALVYHRRRGLEEALGHLREAYALGEEAHPAPLVLEAISGGSGGSGGSAGMSPIHV
RAHPGDVAERVLLPGDPGRAEWIAKTFLQNPRRYNDHRGLWGYTGLYKGVPVSV
QTTGMGTPSAAIVVEELVRLGARVLVRVGTAGAASSDLAPGELIVAQGAVPLDGT
TRQYLEGRPYAPVPDPEVFRALWRRAEALGYPHRVGLVASEDAFYATTPEEARAW
ARYGVLAFEMEASALFLLGRMRGVRTGAILAVSNRIGDPELAPPEVLQEGVRRMVE
VALEAVLEVLETSLGIYSAQSASGDAANLVEHHHHHH

Mw: 74,517.44 Da **ε280 = 62,340 M^{-1} cm^{-1}**

1.2. Calculation of the phosphorolytic activity of the fusion proteins

To compare the enzymatic activities of a fusion enzyme with its native counterparts, we need to account for the proportion of the fusion enzyme that corresponds to each enzymatic domain. This is necessary because only the mass of the fusion enzyme that pertains to the specific domain should be considered when calculating the activity (in IU/mg enzyme).

TP/PNP I-His Mw: 74.517 KDa ***Tt*TP Mw:** 45.300 KDa ***Tt*PNP I Mw:** 25.415 KDa

Proportion of TP domain (TP_{factor})

TP_{factor} =Mw *Tt*TP/ Mw TP-PNP I-His = 45.300 KDa/ 74.517 KDa = 0.607

Proportion of PNP-I domain (PNP I_{factor})

PNP I_{factor} = Mw *Tt*PNP-I/ Mw TP-PNP I-His = 25.415 KDa/ 74.517 KDa = 0.341

Figures

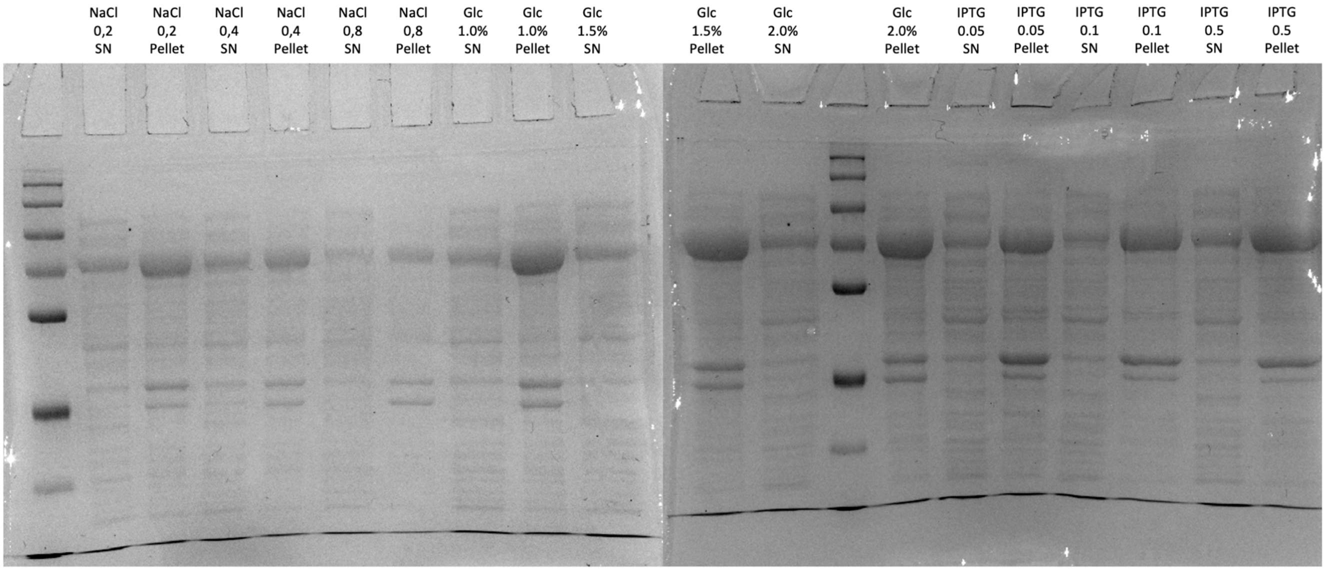

Figure S1. Optimization of protein overexpression using different concentrations of NaCl (0.2 M to 0.8 M), glucose (1% to 2%), or IPTG (0.05 mM to 0.5 mM).

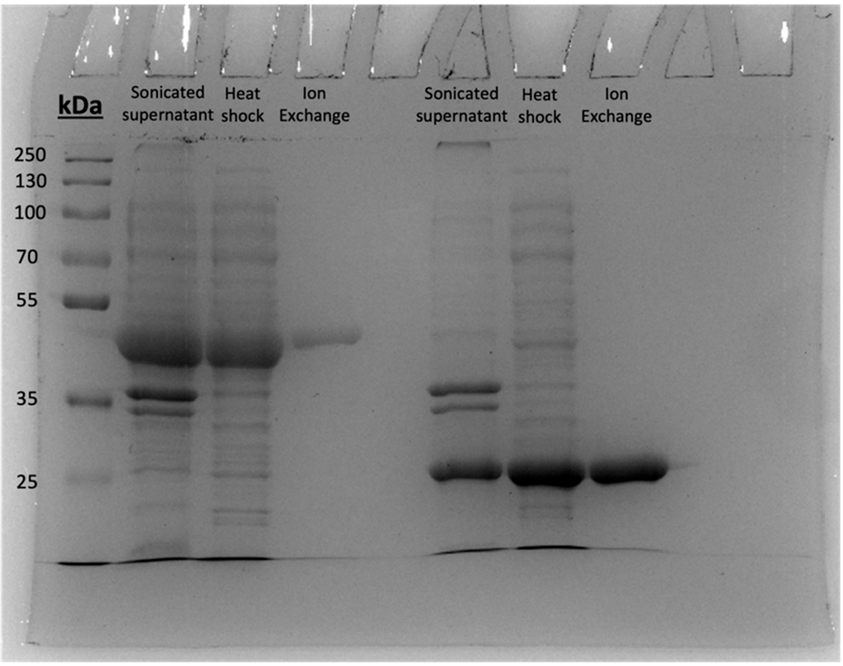

Figure S2. Evaluation of the different purification steps for *Tt*PNP I and *Tt*TP. Lane 1: Markers. Lanes 2-4: Purification process for *Tt*PNP I. Lanes 6-8: Purification process for *Tt*TP

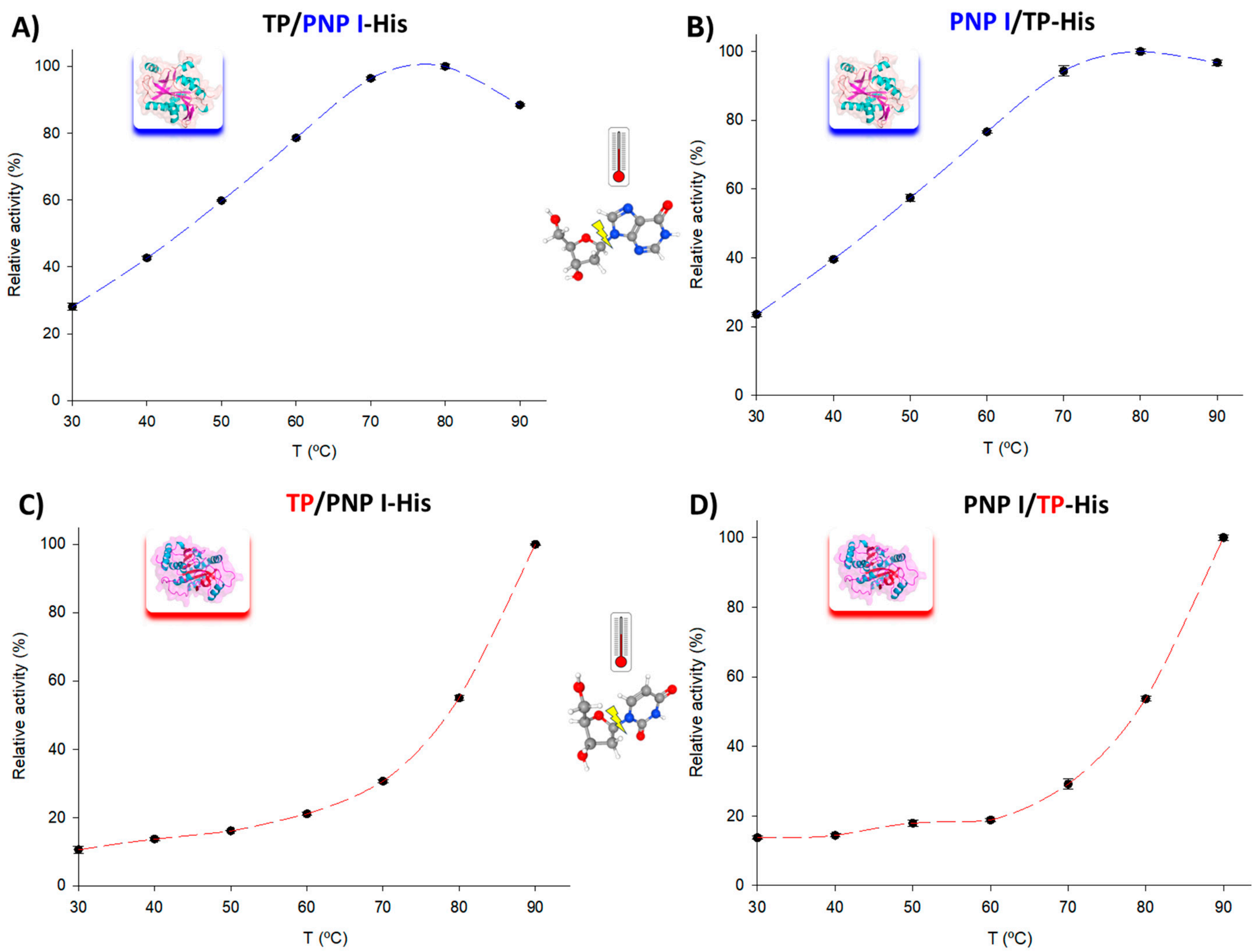

Figure S3. Effect of T on both PNP I domain (2'-deoxyinosine phosphorolysis, blue dotted line) and TP domain (2'-deoxyuridine phosphorolysis, red dotted line) in TP/PNP I-His and PNP I/TP-His fusion enzymes

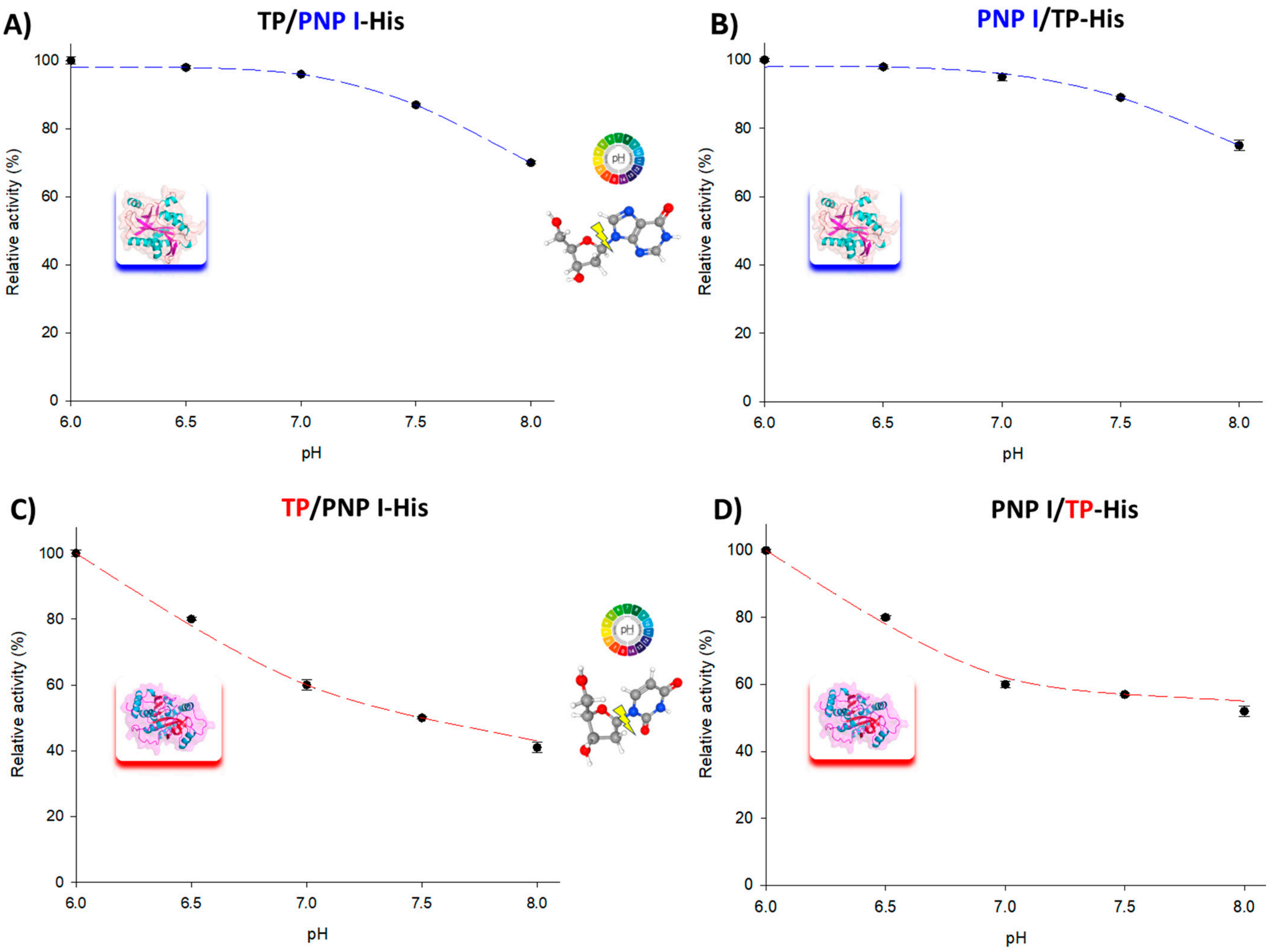

Figure S4. Effect of pH on both PNP I domain (2′-deoxyinosine phosphorolysis, blue dotted line) and TP domain (2′-deoxyuridine phosphorolysis, red dotted line) in TP/PNP I-His and PNP I/TP-His fusion enzymes
